# Nucleotide-decorated AuNPs as probes for nucleotide-binding proteins

**DOI:** 10.1038/s41598-021-94983-y

**Published:** 2021-08-03

**Authors:** Olga Perzanowska, Maciej Majewski, Malwina Strenkowska, Paulina Głowala, Mariusz Czarnocki-Cieciura, Maciej Mazur, Joanna Kowalska, Jacek Jemielity

**Affiliations:** 1 Division of Biophysics, Faculty of Physics, University of Warsaw, Ludwika Pasteura 5, 02-093 Warsaw, Poland; 2 Centre of New Technologies, University of Warsaw, Stefana Banacha 2c, 02-097 Warsaw, Poland; 3 Faculty of Chemistry, University of Warsaw, Ludwika Pasteura 1, 02-093 Warsaw, Poland; 4 Laboratory of Protein Structure, International Institute of Molecular and Cell Biology, Księcia Trojdena 4, 02-109 Warsaw, Poland

**Keywords:** Chemical biology, Materials chemistry, Biophysics, Molecular biophysics

## Abstract

Gold nanoparticles (AuNPs) decorated with biologically relevant molecules have variety of applications in optical sensing of bioanalytes. Coating AuNPs with small nucleotides produces particles with high stability in water, but functionality-compatible strategies are needed to uncover the full potential of this type of conjugates. Here, we demonstrate that lipoic acid-modified dinucleotides can be used to modify AuNPs surfaces in a controllable manner to produce conjugates that are stable in aqueous buffers and biological mixtures and capable of interacting with nucleotide-binding proteins. Using this strategy we obtained AuNPs decorated with 7-methylguanosine mRNA 5’ cap analogs and showed that they bind cap-specific protein, eIF4E. AuNPs decorated with non-functional dinucleotides also interacted with eIF4E, albeit with lower affinity, suggesting that eIF4E binding to cap-decorated AuNPs is partially mediated by unspecific ionic interactions. This issue was overcome by applying lipoic-acid-Tris conjugate as a charge-neutral diluting molecule. Tris-Lipo-diluted cap-AuNPs conjugates interacted with eIF4E in fully specific manner, enabling design of functional tools. To demonstrate the potential of these conjugates in protein sensing, we designed a two-component eIF4E sensing system consisting of cap-AuNP and 4E-BP1-AuNP conjugates, wherein 4E-BP1 is a short peptide derived from 4E-BP protein that specifically binds eIF4E at a site different to that of the 5’ cap. This system facilitated controlled aggregation, in which eIF4E plays the role of the agent that crosslinks two types of AuNP, thereby inducing a naked-eye visible absorbance redshift. The reported AuNPs-nucleotide conjugation method based on lipoic acid affinity for gold, can be harnessed to obtain other types of nucleotide-functionalized AuNPs, thereby paving the way to studying other nucleotide-binding proteins.

## Introduction

Gold nanoparticles (AuNPs) have many distinctive properties that make them particularly useful for sensor development. At proper doses they are biocompatible and not cytotoxic^1^, and their sizes (and therefore other properties) are easily tunable during synthesis^2^. Moreover, they strongly absorb in the visible region of the light spectrum^3^, and most importantly, their surfaces are easily functionalized with various moieties, including biomolecules^4^. One of the key features of surface-functionalized gold-nanoparticles is their ability to undergo controlled aggregation induced by intermolecular interactions, which results in a change in the color of the AuNP solution from deep-red to purple or blue^5^. This phenomenon has been extensively used to develop AuNP-based sensors and molecular probes. AuNP-based colorimetric probes have previously been used for heavy-metal ion detection^6^, imaging protein conformational changes^7^, and studies of biomolecular interactions^8^. Modifying AuNP surfaces with small nucleotides has been shown to provide certain advantages for stability^9,10^. For example, Zhao et al. prepared AuNPs in the presence of ATP as a stabilizing molecule and found that such NPs are more stable in aqueous solution compared to citrate-stabilized AuNPs^10^. We envisaged that nucleotide-decorated AuNPs can also be harnessed to optically sense nucleotide binding proteins, provided that the ligands can be introduced in a controllable manner that is also compatible with biological interactions. In this work, we aimed to test this idea using mRNA 5’ cap analogs as model nucleotides.

m^7^G cap is a nucleotide structure present at the 5'-end of eukaryotic mRNA, and consists of N-7-methylated guanosine connected to an mRNA chain via a 5'–5' triphosphate bridge^11^. Cap takes part in several important cellular processes through multiple interactions with cap-binding proteins^12^, which participate in mRNA maturation, transport, translation and degradation^13,14^. Eukaryotic translation initiation factor 4E (eIF4E) plays a key role in ribosome recruitment during protein biosynthesis^15^, serving as the cap-binding part of the eIF4F pre-initiation protein complex^16^. eIF4E is often overexpressed and up-regulated in cancer cells, therefore it has been identified as a cancer marker and therapeutic target^17,18^. Nathan et al. used Western blot analyses and the immunohistochemical staining of surgical margins of head and neck squamous cell cancers (HNSCCs) to show eIF4E overexpression in margins otherwise defined as lacking histological alterations due to tumor growth^19^. Therefore, the authors postulated that significant molecular changes occur in advance of any histological signs of tumor recurrence, thereby identifying eIF4E as a highly sensitive marker for HNSCC growth. Zhou et al. showed that a relationship exists between eIF4E overexpression, angiogenesis, and vascular invasion of cancer cells^20^. Elevated eIF4E expression was found to correlate with worse overall and disease-free patient survival, which confirmed eIF4E level to be an independent prognostic factor for breast cancer. To date, eIF4E expression levels have been detected either by Western-blot or immunohistochemical methods. Both of these techniques are rather time-consuming and, in the case of immunohistochemistry, requires an invasive procedure to obtain samples. Consequently, the development of novel and fast methods for determining eIF4E concentration levels in biological samples is a crucial objective.

Herein, we describe the synthesis of AuNPs functionalized with eIF4E-binding nucleotides and peptides, and their evaluation as colorimetric molecular probes for the detection and quantification of eIF4E. First, cap-AuNPs were prepared from dinucleotide cap analogs modified with lipoic acid and citrate-stabilized AuNPs in a two-step shell-exchange process. Lipoic acid has been previously used for modification of gold surfaces^21–25^, and other types of (nano)materials^26^, and here we found it shows sufficient chemical stability to be compatible with nucleotide chemistry. We characterized the cap-modified AuNPs for physicochemical and biochemical properties and optimized the cap-ligand density on the AuNP surfaces by the addition of a neutral diluting molecule, which achieved both sufficient AuNP stability and proper recognition by eIF4E. We then investigated the binding of eIF4E by the conjugates using UV–Vis spectroscopy, dynamic light scattering (DLS), and transmission electron microscopy (TEM). In parallel, we modified AuNPs with a small 4E-BP1^27,28^ peptide to create conjugates interacting with eIF4E at a different site to the cap-AuNPs^29,30^. Finally, we used both types of conjugate to create a proof-of-concept colorimetric sensor based on nucleotide-decorated AuNPs, where binding of the protein causes a change visible with a naked eye.

## Results and discussion

### Synthesis and characterization of AuNP-cap analog conjugates

**Figure 1:**
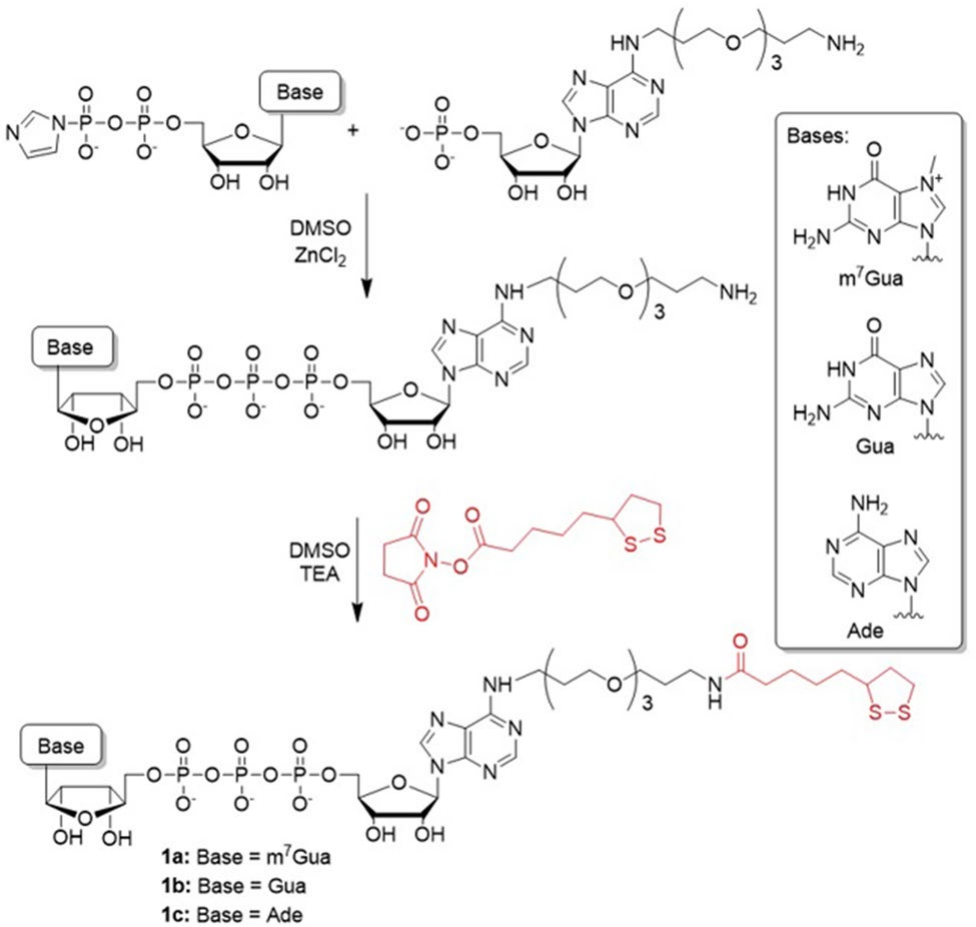
Synthesis of cap analogs modified with the lipoic acid moiety. Compound **1a** is a derivative of the naturally occurring eukaryotic m^7^G cap structure; compounds **1b** and **1c** are non-functional cap analogs used in control experiments with cap-binding protein.

To efficiently functionalize AuNPs, we synthesized dinucleotide cap analogs modified with the lipoic acid moiety, which serves as a molecular anchor with high affinity for gold surfaces^31^ (Fig. 1). Three dinucleotide 5’ cap analogs **1a–1c** were prepared: a naturally occurring cap-0^11^ analog (m^7^GpppA-Lipo, **1a**) and two structurally similar dinucleotides carrying guanine or adenine instead of N^7^-methylguanine (**1b** or **1c**, respectively; Fig. 1). We designed the two latter compounds as non-functional cap analogs (i.e., not recognized by cap-binding proteins) and used them as negative controls in subsequent experiments.

 To obtain the target compounds, an appropriate activated adenosine, guanosine, or 7-methylguanosine 5’-diphosphate was reacted with N6-(1-amino-4,7,10-trioxatridec-13-yl)adenosine 5’-monophosphate. The dinucleotides were then conjugated with the *N*-hydroxysuccinimide (NHS) ester of lipoic acid, purified, and characterized using HPLC, mass spectrometry (MS), and NMR spectroscopy (Supplementary Information, Figures S1–S6).

**Figure 2:**
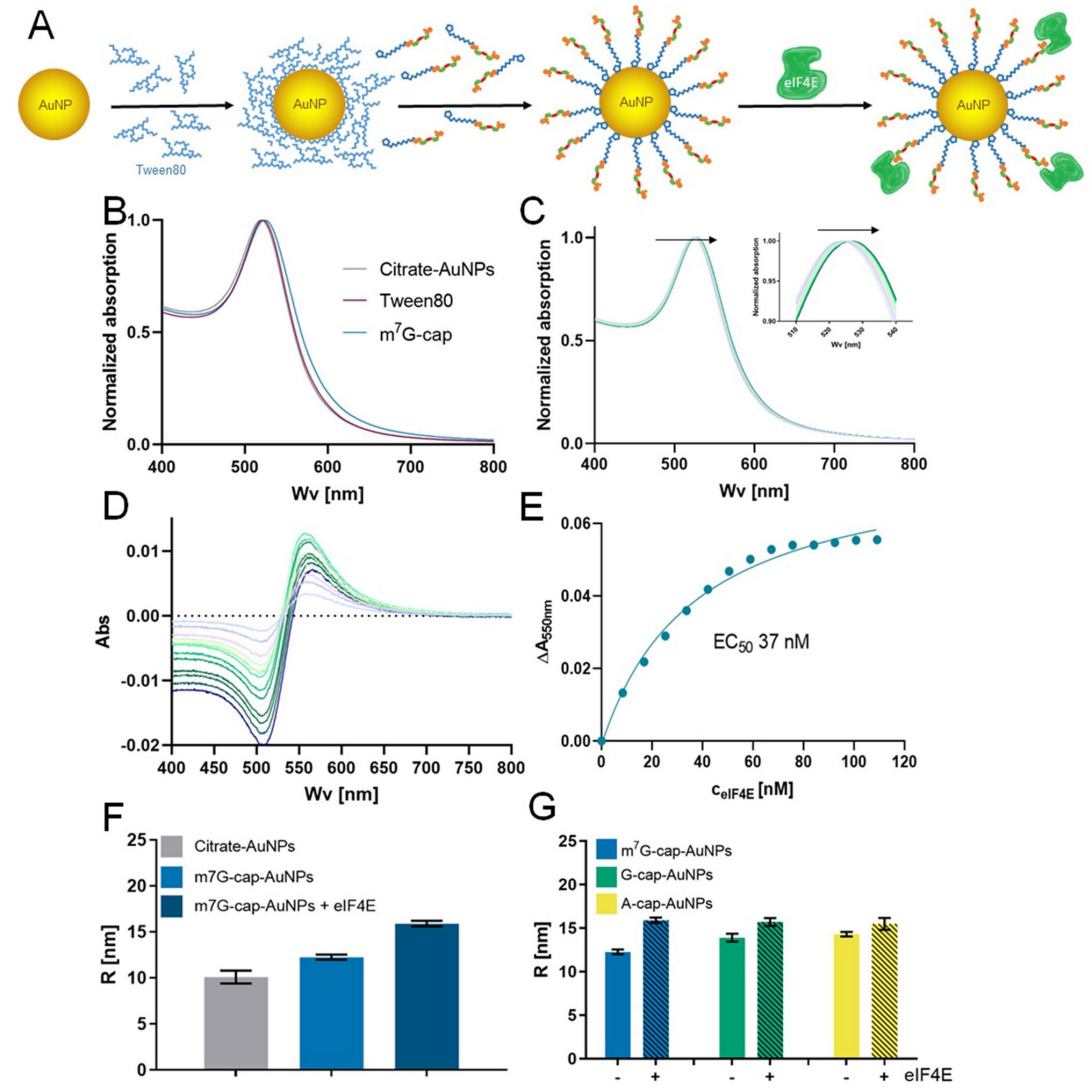
Two-step AuNP surface modification with 5’ cap analogs. **(A)** Citrate AuNPs are incubated with Tween80 overnight before adding the nucleotide solution (**1a**, **1b**, or **1c**) to stabilize the AuNPs during surface modification for further biding experiments with eIF4E. **(B)** Absorption spectra of AuNPs stabilized with citrate, Tween80 or **1a**. **(C)** Normalized absorption spectra obtained by the titration of m^7^G-cap-AuNPs conjugates with eIF4E protein. **(D)** Differential absorption spectra obtained from the same titration experiment. **(E)** Absorption changes at 550 nm for m^7^G-cap-AuNPs as a function of increasing eIF4E concentration. (F) DLS hydrodynamic radii of AuNPs differing in surface composition. **(G)** DLS-determined radii of AuNPs modified with various cap analogs in the presence and absence of eIF4E (150 nM). The concentration of AuNPs in all experiments and at all modification stages was 600 pM, except for DLS measurements (150 pM).

 The lipoic acid moiety was introduced at the N6 position of the adenosine moiety in each compound through a 1,4,7,10-trioxadodecadiamine linker. Citrate-stabilized AuNPs were synthesized by the Turkevich method^32,33^. The particles were close to monodispersed (± 10% size deviation), with diameters of 17.6 ± 1.5 nm determined by TEM or 17.0 ± 1.9 nm as calculated using UV–Vis spectral data and previously reported equations^34^. Our initial attempts to prepare AuNP-cap conjugates by the direct functionalization of citrate AuNPs with cap analogs were unsuccessful due to particle aggregation under the experimental conditions. Hence, we used a two step-protocol, which included modifying the AuNP surfaces with Tween80 (a detergent)^35^, followed by the addition of the cap analog (Fig. 2A). This additional step increased AuNP stability during the surface modification process, which prevented aggregation (Figure S7). The cap-AuNP conjugates were purified by repeated centrifugation and re-suspension in aqueous buffer. The resulting AuNPs had more negative Zeta potential (− 12.3 mV) compared to Tween80-modified AuNPs (− 8.2 mV), were stable for up to one month in water, and did not aggregate or shell-exchange in the presence of non-specific proteins and unmodified nucleotides.

 AuNPs are sensitive to changes in the compositions of their organic encasements, which can be followed by observing shifts in the AuNP absorption band by UV–Vis spectroscopy^36,37^. Our citrate stabilized-AuNPs exhibited an absorption maximum at around 520 nm. Interestingly, increases in the diameter of the monolayer caused by the introduction of different ligands on the surface were manifested in slight but consistent redshift of the AuNPs absorption band (Fig. 2B). Further redshift was observed in the presence of increasing concentrations of eIF4E protein (Fig. 2C). However, the changes in absorption maximum wavelength were too small to enable any reliable functional analysis. In order to best convey the UV–VIS spectral change effect, we have experimentally chosen a different wavelength that exhibited the highest sensitivity to AuNPs surface composition. To that end, the absorption spectra were normalized against the absorption maximum and the wavelength that was most sensitive to the change in monolayer composition was determined by differential absorption spectroscopy (Fig. 2D). All investigated cap-AuNP conjugates exhibited the largest absorption shift at 550 nm and this wavelength was used in subsequent titration experiments directed at UV–VIS monitoring of AuNPs interaction with eIF4E. The absorption changes at 550 nm were plotted as a function of eIF4E yielding a binding curve, from which we determined half maximal effective protein concentration (EC_50_) that reflects the apparent binding affinity of eIF4E for modified AuNPs (Fig. 2E).

The interaction of AuNPs with eIF4E was independently investigated by DLS. First, the size of differently decorated AuNPs was determined. Incubating AuNPs with cap analogs resulted in an increase in the hydrodynamic radius R_H_ (Fig. 2F), suggestive of successful surface exchange. Incubating the m^7^G-cap-AuNP conjugate with saturating concentration of eIF4E protein resulted in a further increase in R_H_ of 3.6 nm, which is comparable to the size of eIF4E molecule^38^, and suggests that the conjugate interacts with eIF4E. The ability of m^7^G cap analogs such as m^7^GpppA to specifically interact with eIF4E relies, to large extent, on cation-π stacking interaction between 7-methylguanosine and two tryptophane moieties present in the cap binding pocket^39^. Consequently, dinucleotide ligands lacking m^7^G moiety have affinities for eIF4E several orders of magnitude lower, thus being good control molecules for verifying the specificity of observed interactions^39^. To verify the specificity of the eIF4E/m^7^G-cap-AuNP interaction, we studied AuNPs conjugates with GpppA (**1b**) and ApppA (**1c**) (G-cap-AuNPs and A-cap-AuNPs, respectively). Unexpectedly, G-cap-AuNPs and A-cap-AuNPs also showed increases in R_H_ in the presence of eIF4E, albeit slightly smaller than that observed for the cap-AuNPs (Fig. 2G). These observations are suggestive of unspecific binding, most likely through electrostatic interactions between negatively charged conjugate surfaces and the positively charged cavities in eIF4E. eIF4E, as and RNA binding protein, carries several positively charged cavities responsible for interaction with negatively charged RNA^39,40^. Very tight packing of the cap analog monolayer on the AuNP surfaces, may favor unspecific attraction of opposite charges over specific cap recognition (which is based on 7-methylguanosine cation-π stacking in addition to salt bridges), therefore impairing differentiation between m^7^G, G, and A caps. As such, we next optimized the AuNPs surface composition in favor of specific binding.

### Optimizing the cap-modified conjugate for specific recognition by eIF4E

**Figure 3:**
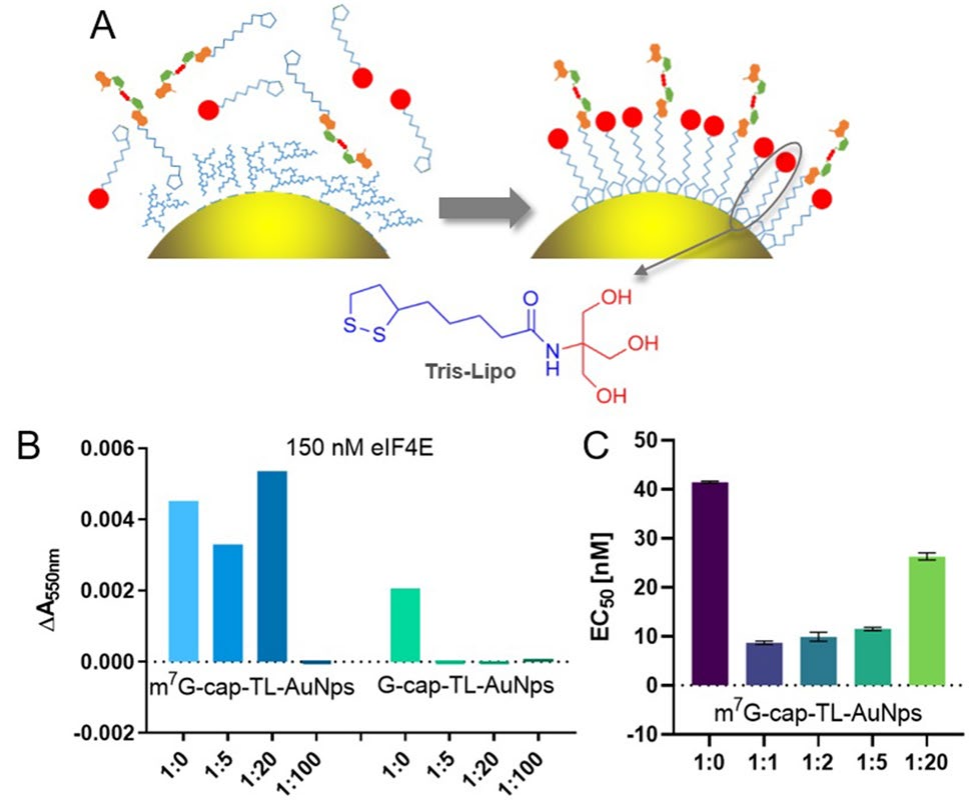
Conjugates carrying mixed envelopes of cap analog **1a** and Tris-Lipo specifically interact with eIF4E. **(A)** Diluting an m^7^G cap monolayer by small Tris-Lipo moieties. **(B)** Absorption changes in the presence of 150 nM eIF4E at 550 nm for m^7^G-cap-TL-AuNP and G-cap-TL-AuNP conjugates “diluted” with Tris-Lipo to various degrees. **(C)** Comparison of EC_50_ coefficients obtained upon titrarion of conjugates solution with eIF4E protein, for different dilutions of m^7^G cap in AuNPs monolayer. The binding curves are shown in Figure S9. The concentration of AuNPs in all experiments was 600 pM.

To reduce the ligand loading on AuNP surface, we designed an improved m^7^G-cap-TL-AuNP conjugate (TL = Tris-Lipo; Fig. 3), in which the cap analog monolayer was “diluted” with a polar, uncharged small molecule of similar affinity for nanogold surfaces (Fig. 3A). The mixed surface composition was achieved by adding Tris-Lipo at a specified ratio relative to cap (1:1, 1:2, 1:5, 1:20, or 1:100) during the second conjugate-preparation step (Figs. 2A, 3A). The attachment of the cap to AuNPs surface under these conditions was confirmed by Fourier-transform infrared spectroscopy in conjunction with attenuated total reflection sampling technique (ATR-FTIR; Figure S8A). Reference G-cap-AuNPs were synthesized in a similar way. The abilities of the various conjugates to interact with eIF4E were then analyzed by UV–Vis spectroscopy, taking advantage of the redshift of the cap-AuNP absorption band that occurs upon eIF4E binding. Changes in absorption at 550 nm were observed in the presence of 150 nM eIF4E for all m^7^G-cap-TL-AuNP conjugates, except for the most dilute ones (Fig. 3B), which suggests that the presence of Tris-Lipo does not prevent interactions with eIF4E. Importantly, the absorption properties of the diluted G-cap-TL-AuNPs were not affected by the presence of eIF4E, regardless of the nucleotide to Tris-Lipo ratio (Fig. 3B). Hence, we concluded that cap analog dilution on the AuNP surfaces by means of Tris-Lipo is a good approach for removing unspecific binding effects.

A series of titration experiments was then conducted in order to compare the relative affinities of variously diluted conjugates for eIF4E; EC_50_ values were determined by titrating the conjugates against eIF4E concentration and plotting changes in absorption at 550 nm (of the normalized spectra) as functions of eIF4E concentration (Fig. 3C, for binding curves see Figure S9A). The lowest EC_50_ value was obtained for conjugates prepared at a 1:1 cap:Tris-Lipo molar ratio, probably due to optimal exposure of the cap moiety to the solution. These results were fairly reproducible, and no AuNP aggregation was observed, which indicates that m^7^G-cap-TL-AuNP stability was not significantly affected by dilution with Tris-Lipo. Hence, these conjugates were identified as optimal and used in all subsequent experiments (and referred to as “m^7^G-cap-TL-AuNPs” from here onwards). As negative controls, the 1:1 m^7^G-cap-TL-AuNP conjugate was titrated against an unspecific protein (BSA; Figure S9A) and the 1:1 G-cap-TL-AuNPs were titrated against eIF4E (Figure S9B); both experiments revealed significantly weaker interactions (EC_50_ for BSA could not be measured due to the very weak response).

**Figure 4:**
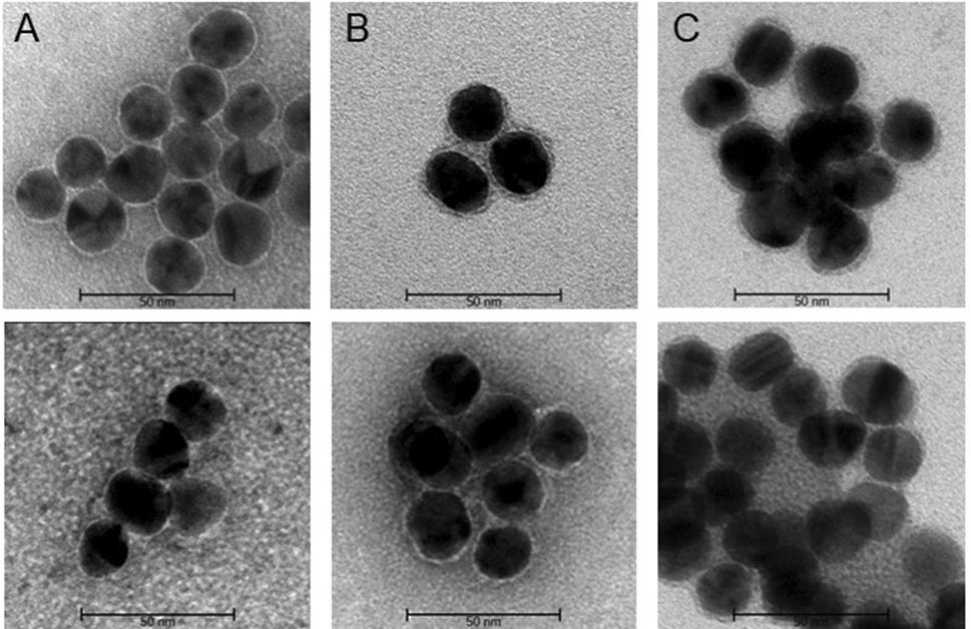
TEM images of 1:1 m^7^G-cap-TL-AuNPs conjugates **(A)** without eIF4E protein, **(B)** incubated with 50 nM concentration of eIF4E, **(C)** incubated with 120 nM concentration of eIF4E. All samples were stained with 1% uranyl acetate solution prior to microscopy. A distinctive halo around gold nanoparticles of roughly the same dimensions as the eIF4E molecule (~ 3.6 nm) can be seen in the images of samples containing protein bound to conjugate surfaces. The concentration of AuNPs in all experiments was 600 pM.

TEM coupled with uranyl acetate staining was used to additionally confirm the ability of the 1:1 m^7^G-cap-TL-AuNP conjugate to bind to eIF4E^41^. TEM images in the presence of eIF4E revealed the formation of a distinctive ~ 3 to 4-nm-thick halo around the conjugate, whereas no such formation was observed in the absence of protein (Fig. 4). Since the thickness of the halo is similar to the diameter of eIF4E (3.6 nm)^38^, we believe the halo is representative of eIF4E directly bound to the conjugate. This approach to visualizing AuNP-protein interactions can be further developed to study the formation of protein complexes and to validate inhibitors.

### Preparing and optimizing 4E-BP1 peptide-AuNP conjugates

**Figure 5:**
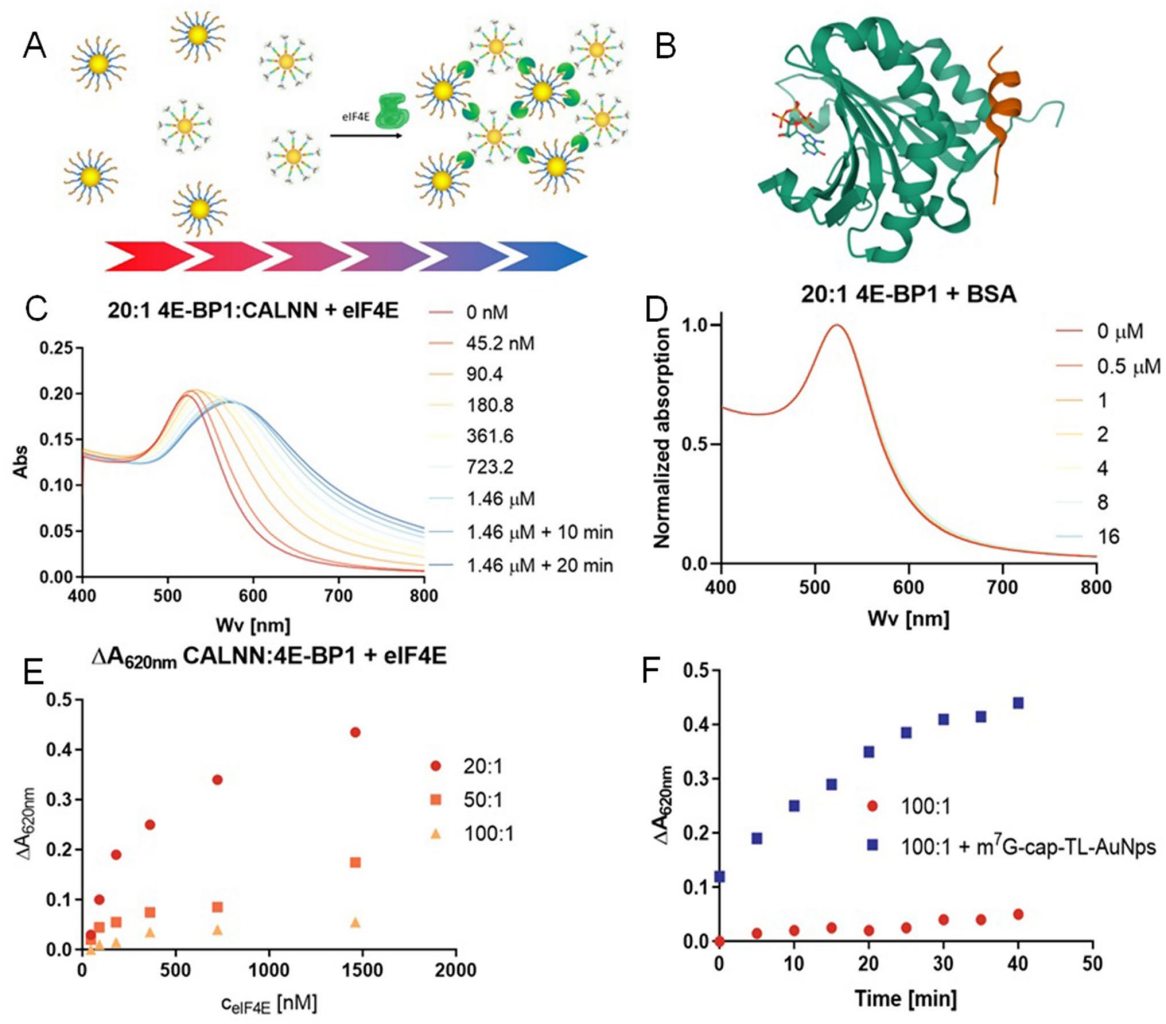
Optimizing the 4E-BP1 conjugate for a two-component AuNP-based eIF4E sensor. **(A)** The design of the two component sensing system that enables the controlled aggregation of AuNP conjugates in the presence of eIF4E. **(B)** The eIF4E molecule in a m^7^GTP/4E-BP1 peptide complex (PDB ID:2V8W)^44^. Coding: eIF4E, green; 4E-BP1, red; nucleotide, balls and sticks. **(C)** Representative changes in the absorption spectra of the 4E-BP1-AuNP conjugate when titrated with eIF4E protein. **(D)** Titration of the same conjugate with unspecific protein (BSA). **(E)** Diluting the 4E-BP1 peptide on the surfaces of the gold nanoparticles results in a decrease in undesired aggregation in the presence of eIF4E. Absorption changes were measured at point of the largest absorption change (620 nm). **(F)** Aggregation rates upon addition of eIF4E protein for the solo 4E-BP1 conjugate and that mixed with m^7^G-cap-TL-AuNPs. The concentration of AuNPs in all experiments was 600 pM.

The m^7^G-cap-TL-AuNP/eIF4E interaction was detectable by UV–Vis spectroscopy; however, binding did not induce AuNP aggregation and, hence, the observed changes in absorption properties were too small to robustly detect eIF4E. Therefore, to create a sensor that undergoes controlled aggregation, we designed a two component AuNPs assay that relies on the use of two types of AuNP conjugate, both capable of interacting with eIF4E protein at two separate binding sites (Fig. 5A)^42^. To that end, we used the 1:1 optimized m^7^G-cap-TL-AuNPs (above) and synthetized peptide-modified AuNPs that target the 4E-BP1/eIF4G binding site in eIF4E^30^. After preliminarily screening several eIF4G- and 4E-BP1-derived peptides (data not shown), we selected a peptide derived from the 4E binding protein (4E-BP1) modified with cysteine at its N-terminus. 4E-BP1 is a translation initiation repressor^29,30^ that binds to eIF4E and inhibits its ability to form the eIF4F translation initiation complex^43^. The selected 4E-BP1 peptide forms a helical structure upon binding to eIF4E without interfering with its cap-binding activity^29^ (Fig. 5B).

In this part of the study we also used a short CALNN (Cys–Ala–Lys–Asn–Asn) peptide sequence as a diluting molecule and AuNP-stabilizing agent^45^, with the thiol group of the terminal cysteine acting as the source of affinity for the AuNP surface. By applying identical biding methods for both stabilizing and functional molecules, we aimed to ensure better control over their actual concentration ratios on the AuNP surfaces. The two-step preparation of the 4E-BP1-AuNP conjugate included modifying the AuNPs with CALNN by overnight incubation, followed by the addition of 4E-BP1 (at a 20:1 CALNN:4E-BP1 molar ratio) and incubation for another 24 h. Titration experiments involving the 4E-BP1-AuNP conjugate and eIF4E revealed significant AuNP aggregation, which proceeded over time and did not produce a stable signal at any timepoint (Fig. 5C).

In control experiments using the 4E-BP1-AuNP conjugates and BSA protein such aggregation was not observed (Fig. 5D), suggesting that the phenomenon is related specifically to instability of eIF4E-4E-BP1-AuNP complexes. The instability effect was mitigated by further diluting 4E-BP1 with CALNN (to a 1:100 ratio) and by preincubating eIF4E with cap-AuNPs before mixing with 4E-BP1-AuNP. Overall these results led us to conclude that aggregation in the presence of eIF4E is likely to have been induced by conformational changes in eIF4E triggered by 4E-BP1-AuNP conjugate binding, which in turn induced unspecific protein–protein interactions. Thus, 4E-BP1-AuNP conjugates alone were unsuitable for the development of a reliable eIF4E sensor. We finally decided that diluting 4E-BP1 with CALNN to a 100:1 ratio was optimal for two-component sensor development, as this ratio led to the least amount of unspecific interactions and uncontrolled aggregation, while preserving the ability of the conjugate to bind to eIF4E and remain a functional part of the assay (Fig. 5E,F).

### Developing a two component eIF4E sensing system

**Figure 6:**
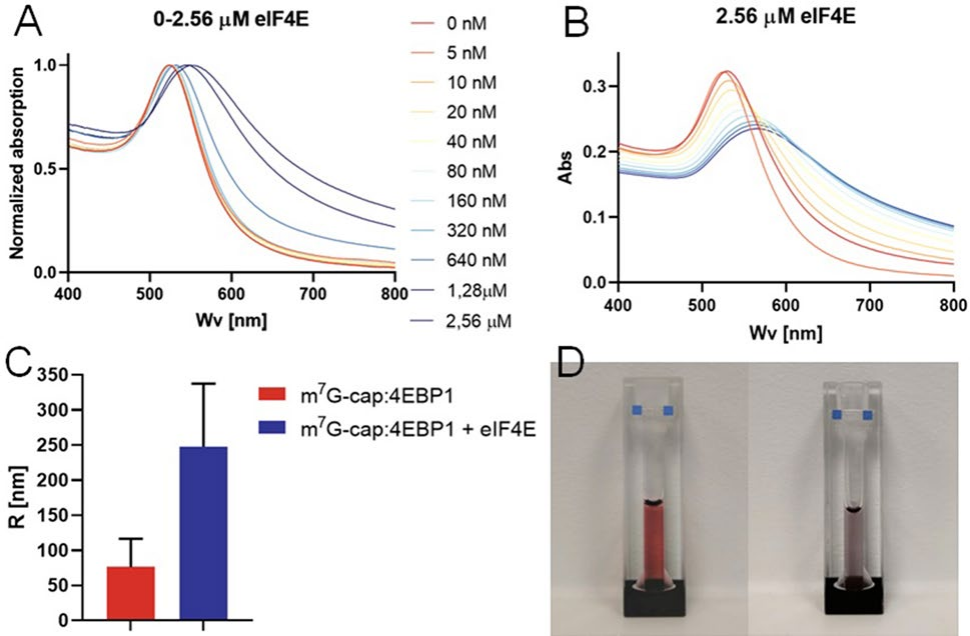
Controlled aggregation experiments with m^7^G-cap-TL-AuNP and 4E-BP1-AuNP conjugates and eIF4E. **(A)** Normalized AuNP absorption band shifts in response to adding increasing concentrations of eIF4E, indicative of conjugate aggregation. Acquired after incubating the conjugates (600 pM) with the protein for 1 h. **(B)** Time-dependence (0 to 55 min) of the conjugate absorption band shift upon addition of a high concentration (2.56 μM) of eIF4E to the conjugate mixture (600 pM). **(C)** DLS hydrodynamic radii of two-component AuNPs solution (150 pM) before and after 1 h incubation with eIF4E (2.56 µM). Data presented as mean ± average SD of n = 3 measurements. Due to non-homogenic character of the conjugate mixtures, high SD values were observed. Size distribution graphs are shown in Figure S13. **(D)** Color of the conjugate mixture (1.2 nM) before (left) and after (right) incubation with eIF4E (2.56 µM) for 2 h.

The AuNPs conjugates selected for two component sensor development were additionally characterized by ATR-FTIR (Figure S8) and Zeta potential measurements. The Zeta potentials were consistent with intended changes of AuNPs surface at corresponding modification stages (Figure S11) and ATR-FTIR confirmed the presence of functional groups characteristic for each ligand in AuNPs conjugates. Next, to verify the functionality of the assay, the m^7^G-cap-TL-AuNP conjugate (0.6 nM) was incubated for 1 h at room temperature with various concentrations of eIF4E, after which an equal volume of the 4E-BP1-AuNP conjugate was added to the mixture to a final concentration of 0.6 nM and incubated for another hour. As a result, the AuNP absorption band shifted toward longer wavelengths, with the most significant shift observed for the m^7^G-cap-conjugate in the presence of the highest concentration of eIF4E (Fig. 6A), which confirmed that both types of conjugate were simultaneously bound by eIF4E. As a result of this eIF4E-mediated “crosslinking”, an event that resembled AuNP aggregation was easily observed—the color of the AuNP solution changed from deep-red to purple-blue (Fig. 6D). We also showed that the UV–Vis spectrum of the AuNPs stabilized after 1 h of final incubation (Fig. 6B), which confirmed a different nature of the observed phenomenon than in the case of 4E-BP1-AuNPs. The limit of detection (LOD) was calculated to be 2.34 nM, although naked eye detection was possible for eIF4E concentration 500 nM and higher (Figure S10C). This result is comparable with other AuNPs-based colorimetric systems designed for protein detection^46,47^. The concentration range for which a linear relationship between absorption shift and eIF4E concentration occurred was 20 to 160 nM (*R*^*2*^ = 0.986) (Figure S10A, S10B). DLS measurements of m^7^G-cap-TL and 4E-BP1-AuNPs mixture were conducted before and after 1 h incubation with eIF4E (2.56 µM) showing great increase in R_H_ (Fig. 6C) and much broader particle size distribution (or polydispersity index PDI) of AuNPs after aggregation occurred, as expected (Figure S14). Changes were even more evident at a higher AuNP conjugate concentration (1.2 nM) and incubation times longer than 2 h at room temperature; however, the conjugates started to precipitate and gather at the bottom of the cuvette under these conditions (Fig. 6D).

A lower initial concentration of the m^7^G-cap-TL-AuNP conjugate resulted in smaller absorption band shifts, as expected (Figure S12A). Control experiments using various mixed conjugate solutions (0.6 nM) and the highest eIF4E concentration were conducted (Figure S12B); introducing the m^7^GTP eIF4E inhibitor to the solution or using G-cap-TL-AuNPs in the assay resulted in significantly smaller absorption band shifts. Similarly, incubating the conjugate solution with BSA protein resulted in a very small absorption band redshift (Figure S13A). Incubating the same conjugate mixture with both eIF4E and BSA showed that BSA caused slight increase in absorption shift, probably due to stabilization of the recombinant eIF4E protein in the presence of BSA, but the increase was similar for three very different BSA concentrations (80 nM, 800 nM and 8 µM) (Figure S13B, S13C). This indicates no specific interactions between BSA and AuNPs conjugates occurred.

**Figure 7:**
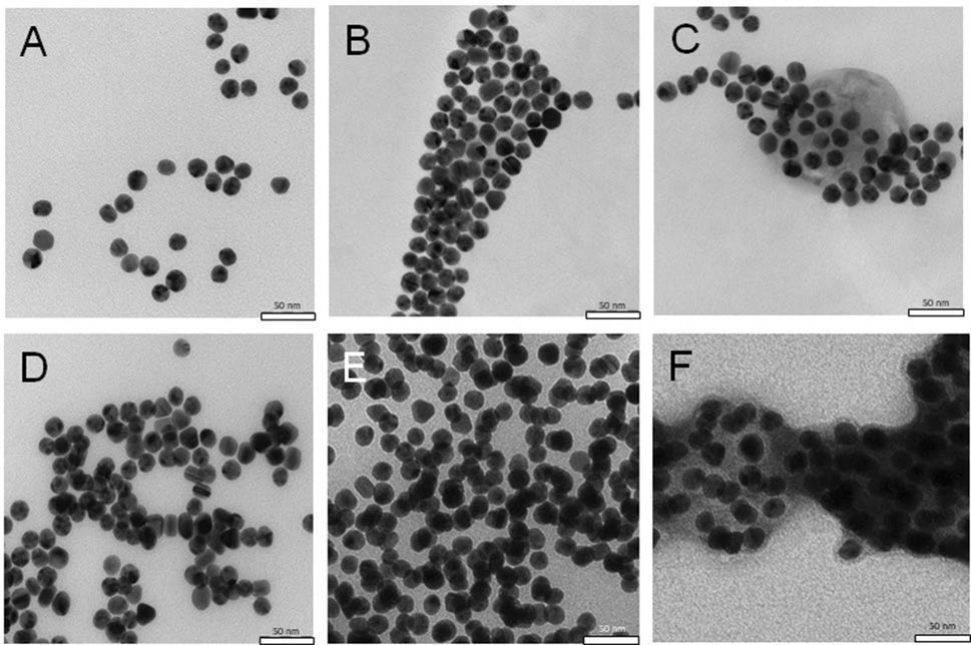
TEM images of m7G-cap-TL-AuNP and 4E-BP1-AuNP conjugates with eIF4E protein. **(A)** m7G-cap-TL-AuNP conjugate. **(B)** 4E-BP1-AuNPs conjugate. **(C)** Mixture of m7G-cap and 4E-BP1 conjugates. **(D)** Mixture of m7G-cap and 4E-BP1 peptide conjugates after incubating for 1 h with eIF4E (2.56 µM). The formation of the first AuNP aggregates is visible. **(E)** Mixture of m7G-cap and 4E-BP1 conjugates after incubation for 2 h with eIF4E. **(F)** TEM images of the same conjugate mix incubated for 2 h with 2.56 μM concentration of eIF4E, and stained with 1% uranyl acetate to visualize the organic layer. The concentration of AuNPs in all experiments was 600 pM.

To further verify the hypothesized working mode of the assay, both types of AuNP conjugate, as well as their mixture were subjected to TEM (Fig. 7). Solo m^7^G-cap-TL-AuNPs and solo 4E-BP1-AuNPs formed uniform clusters (Fig. 7A,B, respectively); the mixture of both conjugate types also showed similar properties (Fig. 7C).

When incubated in the presence of eIF4E, the mixture of both conjugate types gradually aggregated (Fig. 7D,E), with aggregates already visible after 1 h of incubation, but individual nanoparticles were also still observed. Longer incubation (2 h) resulted in the formation of much larger, unstructured aggregates. Staining with 1% uranyl acetate prior to microscopy enabled the visualization of the organic layer surrounding conjugates (Fig. 7F). Due to high protein concentration and the presence of peptides on AuNPs surface it was not possible to clearly visualize individual protein molecules. It is also important to mention the high tendency of nanoparticles in suspension to form artifacts upon drying while preparing TEM samples^48^. Thus, presented TEM images are considered supplemental to more meaningful UV–Vis and DLS measurements.

## Conclusions

In this study, we demonstrated an approach that enables AuNP surfaces to be modified with nucleotides in a controllable, adjustable, and functionality-compatible manner, and relies on the use of lipoic-acid-modified nucleotides. The utility of this approach was demonstrated by modifying AuNPs with 7-methylguanine RNA cap analogs, which are specific binders of the eIF4E oncogenic translation factor. The abilities of these conjugates to interact with eIF4E was confirmed by UV–Vis, DLS, and TEM studies. The properties of the obtained m^7^G-cap-AuNPs were additionally optimized by employing the Tris-Lipo molecule as a cap analog/diluting agent to maximize the specificity of the AuNP/eIF4E interaction. The conjugates were then combined with optimized AuNPs previously modified with the 4E-BP1 eIF4E-specific peptide (4E-BP1-AuNPs) to develop a two component sensing system that enabled UV–Vis and naked-eye eIF4E detection.

Overall, our study highlighted the potential utility of AuNPs in research on cap-binding proteins, taking advantage of the exceptional sensitivities of their UV–Vis absorption properties to changes in monolayer size and composition, as well as intermolecular aggregation. These properties, combined with the potential use of cap-functionalized AuNPs for imaging proteins and protein complex assemblies by TEM, can be harnessed to create novel tools for eIF4E detection and eIF4E inhibitor discovery. The developed approach can be also adjusted to other cap-binding proteins or even de-capping enzymes, provided that properly designed hydrolysis-resistant compounds are used to functionalize AuNPs. The reported AuNP-modification method based on the lipoic acid moiety serving as a molecular anchor with affinity for gold, can be used to obtain other types of nucleotide-functionalized AuNPs, thereby paving the way to studying other nucleotide-binding proteins using similar approaches. Finally, the concept of protein-mediated crosslinking between two types of conjugate can be also adopted to other proteins that bind multiple ligands or allosterically regulated proteins.

## Methods

### General information

Lipoic acid and Tris were purchased from Sigma-Aldrich, and all other reagents and solvents were procured from commercial sources. Reactions involving microwave radiation were conducted in 10-mL heavy-walled glass pressurized vials and snap-on caps. Microwave heating was performed in a CEM Discover single-mode microwave cavity, in dynamic power mode with a maximum power of 10 W and at 50 ± 0.5 °C. Reaction mixtures were stirred with a magnetic stir bar during irradiation. Temperature, pressure, and power were monitored during the course of each reaction using the provided software (standard infrared temperature sensor).

Synthesized nucleotides were purified by ion-exchange chromatography on a DEAE-Sephadex A-25 (HCO_3_-form) column. The column was loaded with the reaction mixture and washed through with excess water to remove metal(II) salt/EDTA complexes. Nucleotides were then eluted using a linear gradient of triethylammonium bicarbonate (TEAB) in deionized water. The compounds were isolated as triethylammonium (TEA) salts after evaporation under reduced pressure with repeated additions of ethanol to decompose TEAB. Yields were calculated on the basis of either sample weight or (preferably) optical milliunits (mOD) of the product. Optical units were measured in 0.1 M phosphate buffer (pH 7 or pH 6 for m^7^G nucleotides) at 260 nm. Analytical HPLC was performed on an Agilent Technologies Series 1200 instrument using a (RP) Supelcosil LC-18-T HPLC column (4.6 × 250 mm, flow rate 1.3 mL/min) with a linear 0–50% methanol gradient in 0.05 M ammonium acetate buffer (pH 5.9) in 7.5 min, or 0–25% methanol in 0.05 M ammonium acetate buffer (pH 5.9) in 15 min, with UV-detection at 260 nm and fluorescence detection (excitation at 280 nm and detection at 337 nm or 450 nm). Semi-preparative HPLC was performed on the same instrument equipped with a Discovery RP Amide C-16 HPLC column (25 cm × 21.2 mm, 5 μm, flow rate 5.0 mL/min) with linear 0–100% acetonitrile gradients in 0.05 M ammonium acetate buffer (pH 5.9) in 120 min, with UV-detection at 260 nm and fluorescence detection (excitation at 280 nm and detection at 450 nm).

The structures and homogeneities of the synthesized nucleotides were further confirmed by mass spectrometry using negative electro-spray ionization and ^1^H and ^31^P NMR spectroscopy. Mass spectra were recorded on a Micromass QToF 1 MS instrument, and NMR spectra were recorded at 25 °C on a Varian Inova 400 spectrometer at 399.94 MHz and 161.90 MHz for ^1^H and ^31^P NMR respectively. ^1^H NMR shifts were calibrated in D_2_O against TMSP as the internal standard, while ^31^P NMR shifts were calibrated against H_3_PO_4_ as the internal standard. Absorption spectra of the nucleotides were recorded on a Shimadzu UV-1800 instrument in the 220–440 nm range with 1 nm resolution.

Starting materials: AMP^PEG13^^49^, m^7^GDP-Im, ADP-Im, and GDP-Im^50^ were synthesized by previously described methods or with minor modifications.

### Chemical syntheses

#### m^7^GpppA^PEG13^

The sodium salt of m^7^GDP-Im (253 mg, 0.46 mmol) and the triethylammonium salt of AMP^PEG13^ (400 mg, 0.61 mmol) were mixed with ZnCl_2_ (734 mg, 5.40 mmol) in DMSO (7.9 mL). The mixture was microwave heated (T = 50 °C, P = 5 W) in 20 min intervals until complete conversion to the product was observed by analytical HPLC. The reaction was terminated by the addition of a solution of EDTA (2.00 g, 5.38 mmol) and NaHCO_3_ (1.00 g, 11.9 mmol) in water (72 mL). The reaction product was purified using ion-exchange chromatography on DEAE-Sephadex. The collected eluate was evaporated to afford 3 750 mOD of m^7^GpppA^PEG13^ as a solid contaminated with ZnCl_2_. The product was repurified using semi-preparative RP HPLC. The collected eluate was lyophilized to afford 236 mg (0.23 mmol) of m^7^GpppA^PEG13^ as the sodium salt. Yield: 50%.

#### GpppA^PEG13^

The sodium salt of GDP-Im (116 mg, 0.22 mmol) and the triethylammonium salt of AMP^PEG13^ (150 mg, 0.23 mmol) were mixed with ZnCl_2_ (219 mg, 1.61 mmol) in DMSO (4 mL). The mixture was microwave heated for 2.5 h and terminated by the addition of a solution of EDTA (599 mg, 1.61 mmol) and NaHCO_3_ (300 mg, 3.57 mmol) in water (20 mL). The reaction product was purified by ion-exchange chromatography on DEAE-Sephadex. The collected eluate was evaporated to afford 2 536 mOD (94.3 μmol) of GpppA^PEG13^ as a solid. Yield: 43%.

#### ApppA^PEG13^

The sodium salt of ADP-Im (193 mg, 0.37 mmol) and the triethylammonium salt of AMP^PEG13^ (84.6 mg, 0.13 mmol) were mixed with ZnCl_2_ (164 mg, 1.21 mmol) in DMSO (4 mL). The mixture was microwave heated for 2 h and terminated by the addition of a solution of EDTA (450 mg, 1.21 mmol) and NaHCO_3_ (225 mg, 2.68 mmol) in water (36 mL). The reaction product was purified by ion-exchange chromatography on DEAE-Sephadex. The collected eluate was evaporated to afford 3 250 mOD (58% purity, 80.6 μmol) of ApppA^PEG13^ as a solid. Yield: 62%.

#### Coupling nucleotides with lipoic acid

The N-hydroxysuccinimide (NHS) ester of lipoic acid was prepared by dissolving lipoic acid in DMSO to a concentration of 0.6 M and adding triethylamine (TEA, 1 equiv.) and *N,N,N′,N*′-tetramethyl-*O*-(N-succinimidyl)uronium tetrafluoroborate (TSTU 1 equiv.), with stirring for 30 min at room temperature. The nucleotide was concurrently dissolved in DMSO to a concentration of 0.1 M, after which the activated lipoic acid was added in 2 equiv. portions to the nucleotide solution in 20 min intervals, until complete conversion to the product was observed by analytical HPLC. The reaction was terminated by tenfold dilution with water and neutralization with acetic acid. The white excess lipoic acid sediment was removed by centrifugation, the reaction product was purified by semi-preparative RP-HPLC, and the collected eluate was lyophilized.

#### 1a. m^7^GpppA^PEG13Lipo^

Lipoic acid (124 mg, 0.603 mmol), TEA (85 μL, 0.607 mmol), and TSTU (183 mg, 0.607 mmol) were dissolved in DMSO (1.01 mL), and the ammonium salt of m^7^GpppA^PEG13^ (28.9 mg, 22.7 μmol) was separately dissolved in DMSO (300 μL). Coupling was performed according to the general procedure, with activated lipoic acid (14 equiv.) added to the reaction mixture. Reaction was terminated after 2 h by the addition of water (6 mL) and the reaction product was purified by semi-preparative RP-HPLC. The collected eluate was lyophilized to afford 6.9 mg (5.61 μmol) of m^7^GpppA^PEG13Lipo^ as a solid. Yield: 20%.

HRMS ESI(−) *m/z* calculated for C_39_H_61_N_11_O_21_P_3_S^2-^ [M–H]^−^ 1176.27032, measured 1176.27079, Δ*m/z* = 0.40 ppm; ^1^H NMR (600 MHz; D_2_O) d ppm 1.30 (2 H; quin; J = 7.6 Hz; L3) 1.44—1.59 (3 H; m; L2; L6”) 1,63 (1 H; dtd; J = 12.4; 7.6; 7.6; 6.2 Hz; L4”) 1.75 (2 H; tt; J = 6.7; 6.3 Hz; P9) 1.86 (1 H; dtd; J = 12.4; 7.6; 7.6; 6.2 Hz; L4’) 1.99 (2 H; quin; J = 6.6 Hz; P2) 2.18 (2 H; t; J = 7.3 Hz; L1) 2.39 (1 H; dq; J = 12.7; 6.2 Hz; L6’) 3.09 (1 H; dt; J = 11.5; 6.2; L7”) 3.14 (1 H; dt; J = 11.5; 6.2; L7’) 3.22 (2 H; t; J = 6,7 Hz; P10) 3.54 (2 H; t; J = 6.3 Hz; P8) 3.58—3.67 (11 H; m; P3; P4; P5; P6; P7; L5) 3.69 (2 H; t; J = 6.6 Hz; P1) 3.99 (3 H; s; m7GH7) 4.24–4.31 (3 H; m; AdH5”; m7GH5’; m7GH5”) 4.34–4.41 (4 H; m; AdH4’; AdH5’; m7GH3’; m7GH4’) 4.49 (1 H; dd; J = 5.3; 3.5 Hz; AdH3’) 4.51 (1 H; dd; J = 5.0; 3.5 Hz; m7GH2’) 4.68 (1 H; dd; J = 5.9; 5.3 Hz; AdH2’) 5.88 (1 H; d; J = 3.5 Hz; m7GH1’) 6.01 (1 H; d; J = 5.9 Hz; AdH1’) 8.20 (1 H; s; AdH2) 8.41 (1 H; s; AdH8) 8.92 (1 H; s; m^7^GH8-*exchangeable*). ^31^P NMR (243 MHz, D_2_O); d ppm − 25.38 (1 P; dd; J = 20.0; 19.1 Hz; Pβ) − 13.89 (1 P; d; J = 20.0 Hz; Pα) − 13.77 (1 P; d; J = 19.1 Hz; Pγ).

#### 1b. GpppA^PEG13Lipo^

Lipoic acid (206 mg, 1.0 mmol), TEA (142 μL, 1.01 mmol), and TSTU (301 mg, 1.01 mmol) were dissolved in DMSO (1.69 mL), and the triethylammonium salt of GpppA^PEG13^ (83 mg, 65 μmol) was concurrently dissolved in DMSO (500 μL). Coupling was performed according to the general procedure, with activated lipoic acid (10 equiv.) added to the reaction mixture. The reaction was terminated after 3 d by the addition of water (11 mL) and the reaction product was purified by semi-preparative RP-HPLC. The collected eluate was lyophilized to afford 16.6 mg (13.7 μmol) of GpppA^PEG13Lipo^ as a solid. Yield: 21%. HRMS ESI(−) *m/z* calculated for C_38_H_59_N_11_O_21_P_3_S_2_^−^ [M–H]^−^ 1162.25467, measured 1162.25328, Δ*m/z* = 1.20 ppm; ^1^H NMR (400 MHz; D_2_O) d ppm 1.29 (2 H; quin; J = 7.6 Hz; L3) 1.42–1.60 (3 H; m; L2; L6”) 1,62 (1 H; dtd; J = 12.4; 7.6; 7.6; 6.1 Hz; L4” ) 1.75 (2 H; tt; J = 6.6; 6.3 Hz; P9) 1.85 (1 H; dtd; J = 12.4; 7.6; 7.6; 6.1 Hz; L4’) 1,99 (2 H; quin; J = 6.4 Hz; P2) 2.17 (2 H; t; J = 7.2 Hz; L1) 2,38 (1 H; dq; J = 12.7; 6.1 Hz; L6’) 3.04—3.18 (2 H; m; L7’; L7”) 3.22 (2 H; t; J = 6.6 Hz; P10) 3,53 (2 H; t; J = 6.3 Hz; P8) 3.58–3.75 (13 H; m; P1; P3; P4; P5; P6; P7; L5) 4.18–4.33 (5 H; m; AdH5’; AdH5”; GH4’; GH5’; GH5”) 4.33–4.39 (1 H; m; AdH4’) 4.48 (1 H; t; J = 4.5 Hz; GH3’) 4,52 (1 H; t; J = 4.7 Hz; AdH3’) 4,64 (1 H; dd; J = 5.2; 4.5 Hz; GH2’) 4.69 (1 H; t; J = 4.9 Hz; AdH2’) 5.81 (1 H; d; J = 5.2 Hz; GH1’) 6,06 (1 H; d; J = 4.7 Hz; AdH1’) 7.98 (1 H; s; GH8) 8,21 (1 H; s; AH2) 8.41 (1 H; s; AH8). ^31^P NMR (162 MHz; D_2_O) d ppm − 22.34 (1 P; t; J = 19.29 Hz; Pβ) −10.84 (2 P; m; Pα Pγ).

#### 1c. ApppA^PEG13Lipo^

Lipoic acid (407 mg, 1.98 mmol), TEA (284 μL, 2.02 mmol), and TSTU (612 mg, 2.03 mmol) were dissolved in DMSO (3.37 mL), and the triethylammonium salt of ApppA^PEG13^ (204 mg, 162 μmol) was concurrently dissolved in DMSO (1.5 mL). Coupling was performed according to the general procedure, with activated lipoic acid (10 equiv.) added to the reaction mixture. The reaction was terminated after 2 d by the addition of water (24 mL). The reaction product was purified by semi-preparative RP-HPLC. The collected eluate was lyophilized to afford 6.6 mg (5.5 μmol) of ApppA^PEG13Lipo^ as a solid. Yield: 3.4%. HRMS ESI(−) *m/z* calculated for C_38_H_59_N_11_O_20_P_3_S_2_^−^ [M–H]^−^ 1146.25976, measured 1146.25823, Δ*m/z* = 1.33 ppm; ^1^H NMR (400 MHz, D2O) d ppm 1.26 (2 H; quin; J = 7.6 Hz; L3) 1.38–1.56 (3 H; m; L2; L6”) 1.58 (1 H; dtd; J = 12.4; 7.6; 7.6; 6.2 Hz; L4”) 1,74 (2 H; tt; J = 6.6; 6.3 Hz; P9) 1.83 (1 H; dtd; J = 12.4; 7.6; 7.6; 6.2 Hz; L4’) 1.95 (2 H; quin; J = 6.5 Hz; P2) 2.15 (2 H; t; J = 7.2 Hz; L1) 2.36 (1 H; dq; J = 12.7; 6.15 Hz; L6’) 3.03–3.16 (2 H; m; L7’; L7”) 3,20 (2 H; t; J = 6.6 Hz; P10) 3.52 (2 H; t; J = 6.3 Hz; P8) 3.57—3.71 (13 H; m; P1; P3; P4; P5; P6; P7; L5) 4.23—4.38 (6 H; m; AH4’; AH5’; AH5”; AdH4’; AdH5’; AdH5”) 4,49 (1 H; t; J = 4.5 Hz; AH3’) 4,51 (1 H; t; J = 4.5 Hz; AdH3’) 4,60 (1 H; t; J = 4.5 Hz; AH2’) 4.65 (1 H; dd; J = 4.7; 4.5 Hz; AdH2’) 6.01 (1 H; d; J = 4.5 Hz; AH1’) 6.03 (1 H; d; J = 4.7 Hz; AdH1’) 8.12 (1 H; s; AH8) 8.12 (1 H; s; AdH8) 8.25 (1 H; s; AH2) 8,31 (1 H; s; AdH2). ^31^P NMR (162 MHz; D2O) d ppm − 22.32 (1 P; t; J = 19.00 Hz; Pβ) − 10.97 (1 P; d; J = 19.00 Hz; Pα) −10.87 (1 P; d; J = 19.00 Hz; Pγ).

#### Tris-Lipo

The *N*-Hydroxysuccinimide (NHS) ester of lipoic acid was prepared by dissolving lipoic acid (210 mg, 1.02 mmol) in of DMSO (1.7 mL) to a concentration of 0.6 M, after which TEA (140 μL, 1.1 mmol) and TSTU (303 mg, 1.01 mmol) were added and stirred for 30 min at room temperature. Tris(hydroxymethyl)aminomethane (Tris, 1.21 g, 10.0 mmol) was concurrently dissolved in DMSO (3.2 mL), after which the activated lipoic acid was added to the Tris solution in 2 equiv. portions in 20 min intervals. After 24 h, undissolved Tris sediment was removed by centrifugation and DMSO (14 mL) was added to the reaction mixture to a concentration of 50 mM.

#### AuNPs synthesis

HAuCl_4_ · 3H_2_O (4.43 μL, 6.25 μmol) was added to deionized water (25 mL) to a concentration of 0.25 mM. The reaction mixture was heated to reflux with continuous stirring and a 5% aqueous solution of trisodium citrate (214 μL, 43.8 μmol) was added and the mixture was further heated under reflux for 20 min. The reaction was terminated by cooling to room temperature.

### 4E-BP1 peptide solution preparation

The 4E-BP1 peptide sequence was: CCALNN-GG-TRIIYDRKFLMECRNA.

1 mM 4E-BP1 peptide stock solution was prepared by dissolving 4E-BP1 in a 1:1 (v/v) mixture of 2,2,2-trifluoroethanol and PBS buffer (pH 7.2). The stock solution was stored at − 18 °C until required.

#### Preparing AuNP-cap analog conjugates

A gold nanoparticle solution was gently mixed with a Tween80 solution (3.6 mg/mL) in phosphate buffer (10 mM, pH 7.2) in a 1:1 ratio. The mixture was then left at room temperature for at least 1 h to ensure physical adherence of Tween80 to the AuNP surfaces. The AuNP/Tween80 solution (1.9 mL) was then mixed with m^7^GpppA^PEG13Lipo^, GpppA^PEG13Lipo^, or ApppA^PEG13Lipo^ in water (100 μL, 1 mM). To obtain Tris-Lipo dilution, appropriate amount of 50 mM solution of Tris-Lipo in DMSO was mixed with m^7^GpppA^PEG13Lipo^ water solution before mixing with AuNPs to final molar ratio of the ligands of 1:1, 1:2, 1:5, 1:20 or 1:100. The mixture was left for 24 h at room temperature, without stirring. The AuNP solution was shielded from light in every step. Excess ligands and Tween80 were then removed by centrifugation (13,000 rpm, 15 min, 4 °C) and the supernatant was collected. The AuNP-cap analog conjugate was then dissolved in HEPES buffer (50 mM HEPES, 100 mM KCl, 0.5 mM EDTA, pH 7.2) and filtered through a 0.42-µm filter.

#### Preparing the AuNP-4E-BP1 peptide conjugates

A gold nanoparticle solution was gently mixed with a CALNN peptide solution (0.35 mM) in PBS buffer (pH 7.2) at a 10:1 ratio. The mixture was then left at room temperature overnight for the CALNN peptide to physically adhere to the AuNP surfaces. The AuNP/CALNN solution (2 mL) was mixed with a 4E-BP1 stock solution (0.68 µL, 1 mM) and the mixture was left for 24 h at room temperature, without stirring. The AuNP solution was shielded from light. Excess 4E-BP1 and CALNN was removed by centrifugation (13,000 rpm, 15 min, 4 °C) and the supernatant was collected. The AuNP-4E-BP1 conjugate was then dissolved in HEPES buffer (50 mM HEPES, 100 mM KCl, 0.5 mM EDTA, pH 7.2) and filtered through a 0.42-µm filter.

#### Analyzing AuNP-nucleotide-conjugate/eIF4E interactions by UV–Vis spectroscopy

A 0.5 mL aliquot of each conjugate solution (0.6 nM in HEPES buffer) was added into a standard UV–Vis cuvette at room temperature. The conjugate solution was titrated with increasing concentrations of eIF4E protein (0.5 nM to 2.56 µM). After each addition of protein, the mixture was incubated for 3 min at room temperature without stirring, after which the absorption was measured. Absorption spectra were recorded in the 400–800 nm range on the above-mentioned UV-1800 Shimadzu instrument with 0.5 nm resolution.

#### Controlled aggregation assay for detecting eIF4E

The AuNP-cap analog conjugate (0.6 nM in HEPES buffer) was incubated for 1 h at room temperature with various concentrations of eIF4E protein (5 nM to 2.56 µM). An equal volume of the AuNP-4E-BP1 peptide conjugate solution (0.6 nM in HEPES buffer) was then added, and the mixture was incubated at room temperature for another hour, after which absorption was measured.

#### Transmission electron microscopy (TEM)

For the AuNP conjugates, 20 µL of freshly prepared AuNP solution was cast onto a carbon-coated Formvar 300-mesh copper grid and left to dry in air. AuNP TEM images were acquired on a Zeiss Libra 120 EFTEM transmission electron microscope.

#### Transmission electron microscopy (TEM) with uranyl acetate staining

A 600 pM solution of the m7G-cap-TL-AuNP conjugate (incubated with eIF4E protein at 50 nM, 120 nM, or 2.56 µM concentration) was applied to a glow-discharged carbon-coated 300-mesh cooper TEM grid and stained with 1% uranyl acetate. TEM images were acquired with Tecnai T12 BioTWIN 120 kV Electron Microscope (FEI) with acceleration voltage of 120 kV.

#### Dynamic light scattering (DLS)

The particle sizes of the prepared AuNP conjugates were determined by adding 50 µL (0.15 nM of AuNPs in HEPES buffer) of each sample to a standard low-volume DLS cuvette for measurement at 25 °C. The size distribution of each sample was recorded in triplicate using a 658-nm-wavelength laser. The hydrodynamic radii of the AuNP conjugates were measured using a Wyatt DynaPro NanoStar DLS analyzer.

#### Zeta potential

Zeta potentials of AuNP conjugates were determined by adding 1 ml of each sample (0.15 nM of AuNPs in HEPES buffer) to a Zeta deep cell for measurement at 24˚C. Zeta potential of each sample was recorded in triplicate using Zetasizer nano ZS, Malvern.

#### FTIR

FTIR spectra of AuNP conjugates were recorded using ATR sampling technique at room temperature. Samples of ligands used for AuNP surface modifications (Tris-Lipo, m^7^GpppA^PEG13Lipo^, CALNN and 4E-BP1 peptides) were measured as dry powders. AuNP conjugate samples were prepared by centrifugation (13,000 rpm, 15 min), removal of the supernatant containing excess ligand moieties and/or buffer, dissolving the sample in 1 ml of pure water, second centrifugation followed by supernatant removal and lastly by freeze-drying the sample until completely dry. Dry AuNP conjugate powder was transferred directly to the ATR accessory crystal prior to measurement. FTIR spectra were recorded using Shimadzu Fourier Transform Infrared Spectrophotometer IRPrestige-21. Presented spectra are an average of 60 scans.

## Supplementary Information

Supplementary Figures.
