# Supplementary material for: Nucleotide-decorated AuNPs as probes for nucleotide-binding proteins
Source: Sci Rep. 2021 Aug 3;11:15741. doi: 10.1038/s41598-021-94983-y (PMC8333360; doi:10.1038/s41598-021-94983-y)
Supplement: Supplementary file 1 — Supplementary Figures. [file 41598_2021_94983_MOESM1_ESM.docx]

Nucleotide-decorated AuNPs as probes for nucleotide-binding proteins

Olga Perzanowska^1,2^, Maciej Majewski^1^, Malwina Strenkowska^1^, Paulina Głowala^3^, Mariusz Czarnocki-Cieciura^4^, Maciej Mazur^3^, Joanna Kowalska*^1^ Jacek Jemielity*^2^

1 O. Perzanowska, M. Majewski, M, Strenkowska, J. Kowalska*

Division of Biophysics, Faculty of Physics University of Warsaw

Ludwika Pasteura 5, 02-093 Warsaw (Poland)

Email: jkowalska@fuw.edu.pl

2 O. Perzanowska, J. Jemielity*

Centre of New Technologies University of Warsaw

Stefana Banacha 2c, 02-097 Warsaw (Poland)

Email: j.jemielity@cent.uw.edu.pl

3 P. Głowala, M. Mazur

Faculty of Chemistry University of Warsaw

Ludwika Pasteura 1, 02-093 Warsaw (Poland)

4 M. Czarnocki-Cieciura

Laboratory of Protein Structure International Institute of Molecular and Cell Biology, Warsaw

Księcia Trojdena 4, 02-109 Warsaw (Poland)

**Table of contents**

**Figure S1.** HPLC profiles of m^7^GpppA^PEG13Lipo^ (**1a**), GpppA^PEG13Lipo^ (**1b**) and ApppA^PEG13Lipo^ (**1c**) compounds.

**Figure S2.** HRMS ESI (-) TOF profiles.

**Figure S3.** ^1^H NMR spectrum (600 MHz; D_2_O) of m^7^GpppA^PEG13Lipo^ (**1a**).

**Figure S4.** ^1^H NMR spectrum (400 MHz; D2O) of GpppA^PEG13Lipo^ (**1b**).

**Figure S5.** ^1^H NMR spectrum (400 MHz, D2O) of ApppA^PEG13Lipo^ (**1c**).

**Figure S6.** ^31^P NMR spectra of A) m^7^GpppA^PEG13Lipo^ (**1a**) (243 MHz, D2O); B) GpppA^PEG13Lipo^ (**1b**) (162 MHz; D2O); C) ApppA^PEG13Lipo^ (**1c**), (162 MHz; D2O).

**Figure S7.** TEM images of A) citrate-stabilized AuNPs and B) m^7^G-cap-AuNP conjugates.

**Figure S8.** Binding curves obtained by titrating m^7^G-cap-AuNps, G-cap-TL-AuNPs and m^7^G-cap-TL-AuNPs conjugates of different cap to Tris-Lipo ratios against eIF4E.

**Figure S9.** ATR-FTIR spectra.

**Figure S10.** Linear dependence of ΔA_620nm_ on the eIF4E concentration.

**Figure S11.** Zeta potentials of AuNPs at different stages of surface modification.

**Figure S12.** Additional aggregation experiments in two-component solution with eIF4E.

**Figure S13.** Control experiments with BSA protein.

**Figure S14.** DLS-determined size distributions of m^7^G-cap-TL-AuNPs and 4E-BP1-AuNPs 1:1 conjugate mixture.


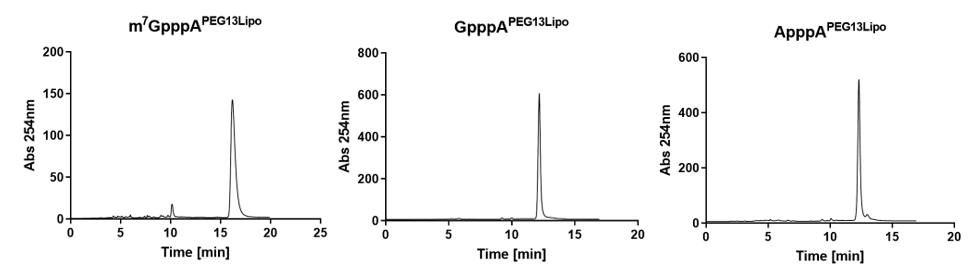
 **Figure S1.** HPLC profiles of m^7^GpppA^PEG13Lipo^ (**1a**), GpppA^PEG13Lipo^ (**1b**) and ApppA^PEG13Lipo^ (**1c**) compounds. Analytical HPLC performed with linear gradient 0–50% of methanol in 0.05 M ammonium acetate buffer (pH 5.9) in 7.5 minutes.


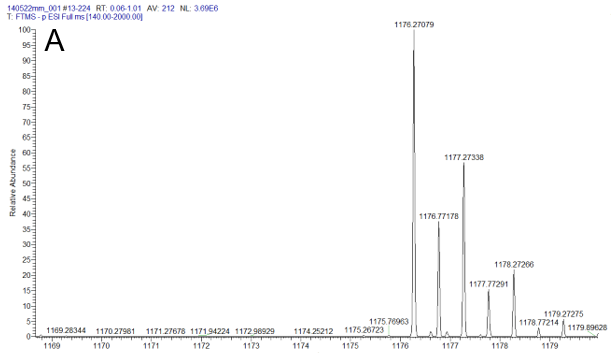

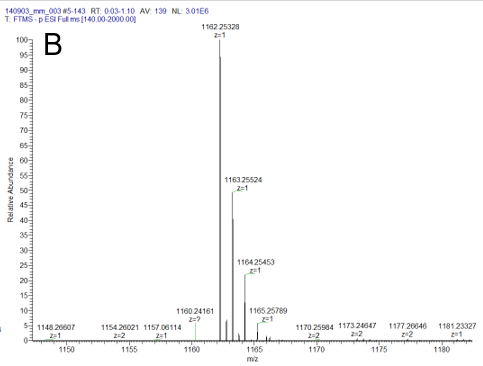


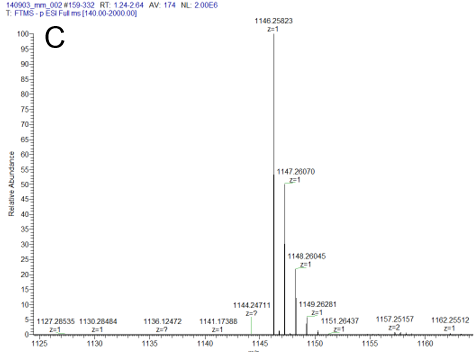


**Figure S2.** HRMS ESI (-) TOF profiles. A) m^7^GpppA^PEG13Lipo^ (**1a)**; B) GpppA^PEG13Lipo^ (**1b**) C) ApppA^PEG13Lipo^ (**1c**).

**Figure S3.** ^1^H NMR spectrum (600 MHz; D_2_O) of m^7^GpppA^PEG13Lipo^ (**1a**).

**Figure S4.** ^1^H NMR spectrum (400 MHz; D2O) of GpppA^PEG13Lipo^ (**1b**).

**Figure S5.** ^1^H NMR spectrum (400 MHz, D2O) of ApppA^PEG13Lipo^ (**1c**).


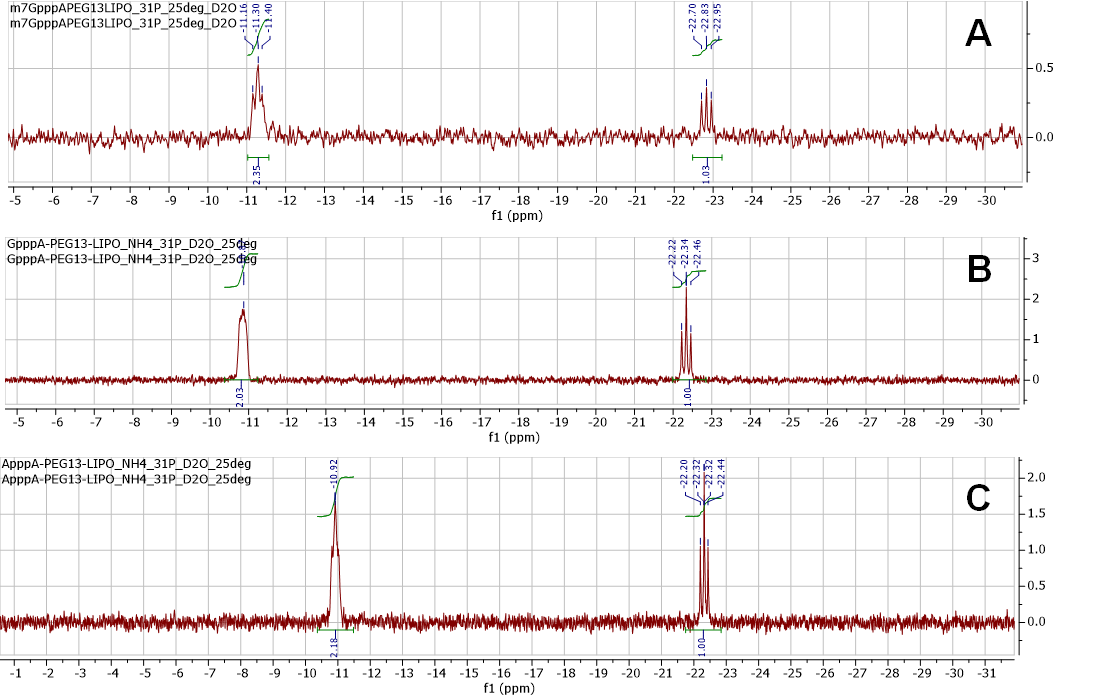


**Figure S6.** ^31^P NMR spectra of A) m^7^GpppA^PEG13Lipo^ (**1a**); B) GpppA^PEG13Lipo^ (**1b**); C) ApppA^PEG13Lipo^ (**1c**).


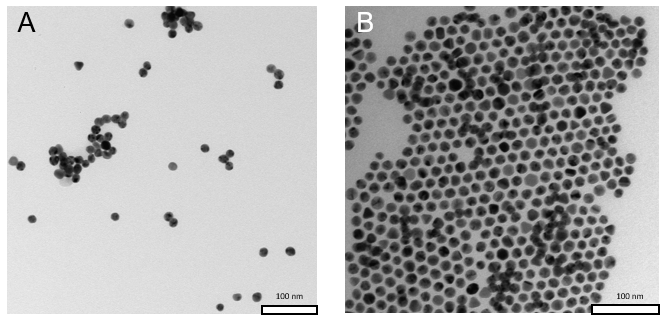


**Figure S7.** TEM images of A) citrate-stabilized AuNPs and B) m^7^G-cap-AuNP conjugates showing that cap-decorated AuNPs are much more stable and less prone to aggregation.


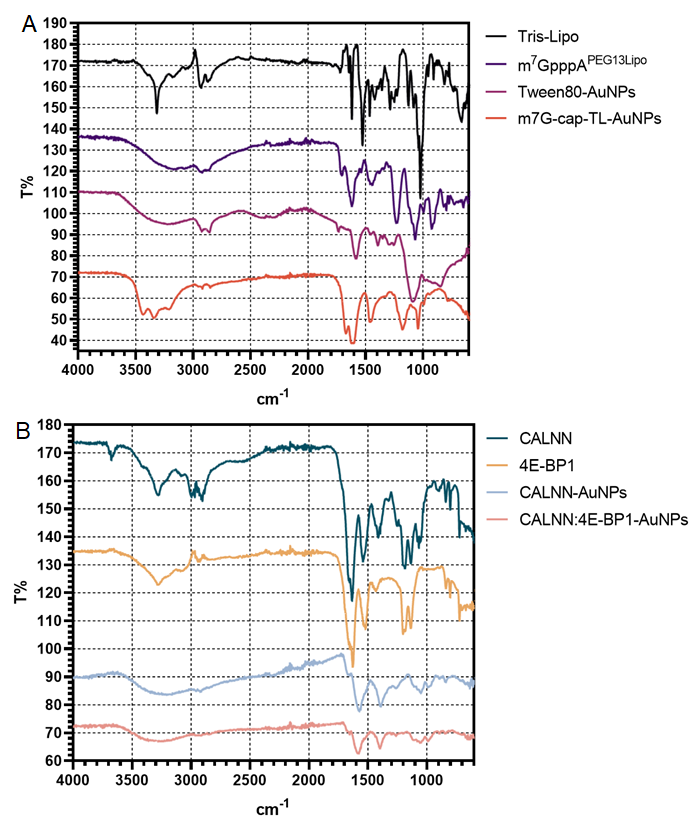


**Figure S8.** ATR-FTIR spectra. A) compilation of FTIR spectra of Tris-Lipo and m^7^GpppaA^PEG13Lipo^ dry ligands, and Tween80-AuNPs and m^7^G-cap-TL-AuNPs. B) compilation of FTIR spectra of CALNN and 4E-BP1 dry peptides, and CALNN-AuNPs and CALNN:4E-BP1-AuNPs.


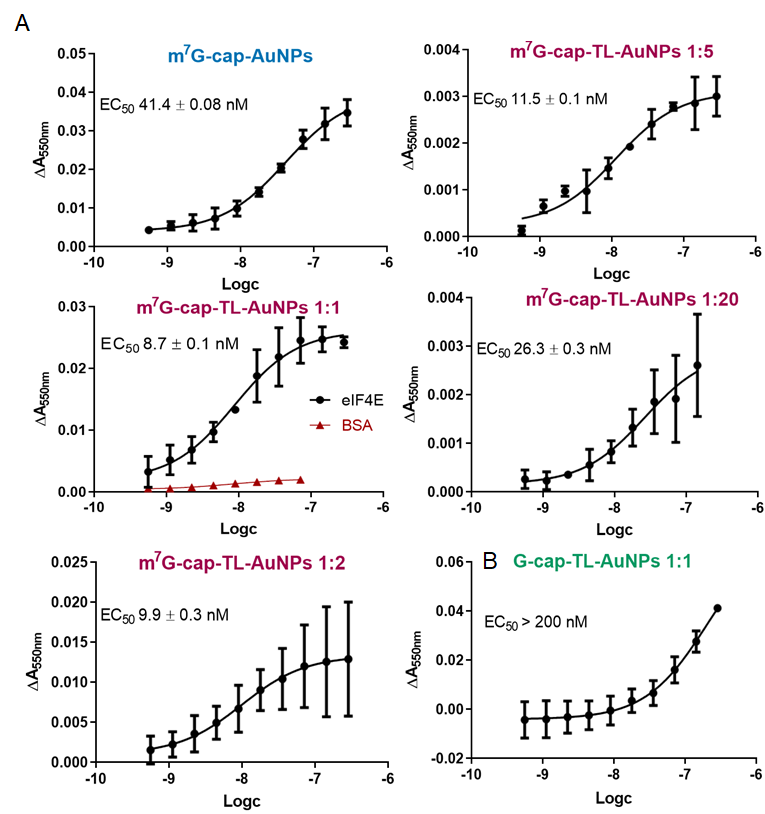


**Figure S9.** Binding curves obtained by titrating m^7^G-cap-AuNps, G-cap-TL-AuNPs and m^7^G-cap-TL-AuNPs conjugates of different cap to Tris-Lipo ratios against eIF4E. EC50 values were determined by plotting changes in absorption at 550 nm as functions of eIF4E concentration A) Binding curves obtained by titrating selected m^7^G-cap-TL-AuNP conjugates against eIF4E or BSA protein. B) Titrating the 1:1 diluted G-cap-TL-AuNP conjugate against eIF4E protein.


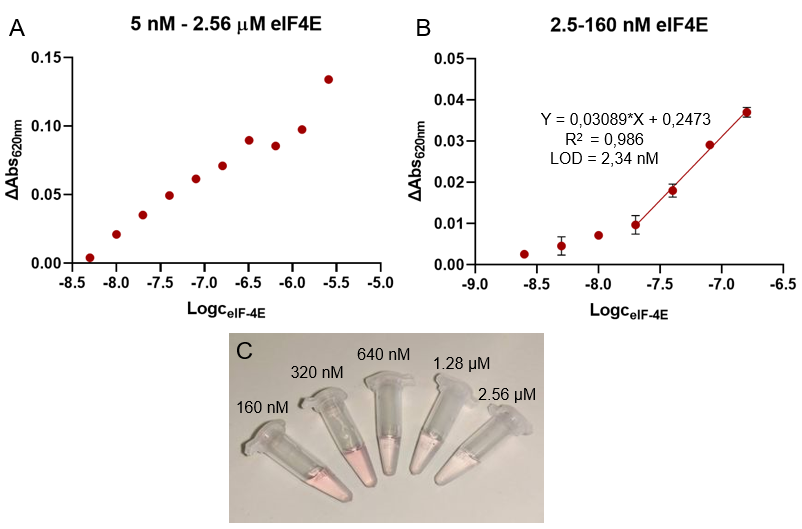


**Figure S10.** Linear dependence of ΔA_620nm_ on the eIF4E concentration. A) eIF4E concentration range between 5 nM and 2.56 µM. B) eIF4E concentration range between 2.5 nM and 160 nM, including linear regression fit. C) AuNPs samples changing color as a result of aggregation.


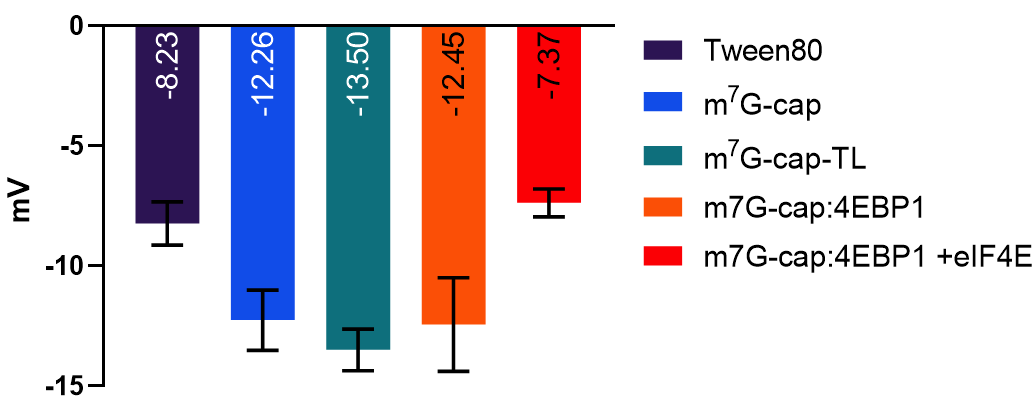


**Figure S11.** Zeta potentials of AuNPs at different stages of surface modification. All measurements were done in HEPES buffer at 24˚C in a Zetasizer nano ZS, Malvern. Data presented as an average ± SD of n=3 independent measurements.


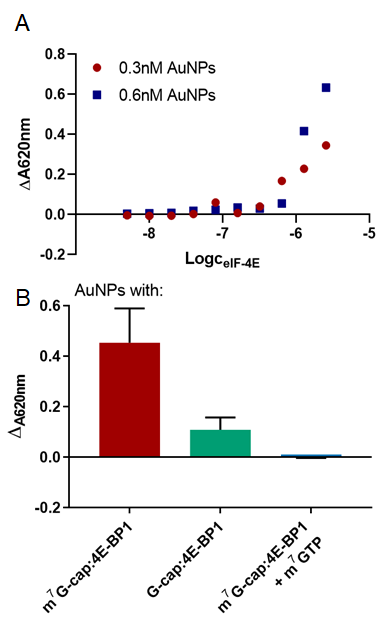


**Figure S12.** Additional aggregation experiments in two-component solution with eIF4E. A) Absorption change for two different concentrations of conjugate in solution; a lower concentration (0.3 nM) decreases the aggregation rate. B) Aggregation rates for control experiments with a eIF4E concentration of 2.56 µM. Inhibition of eIF4E by m^7^GTP as well as a conjugate with an unmethylated cap analog greatly decreases the aggregation rate.


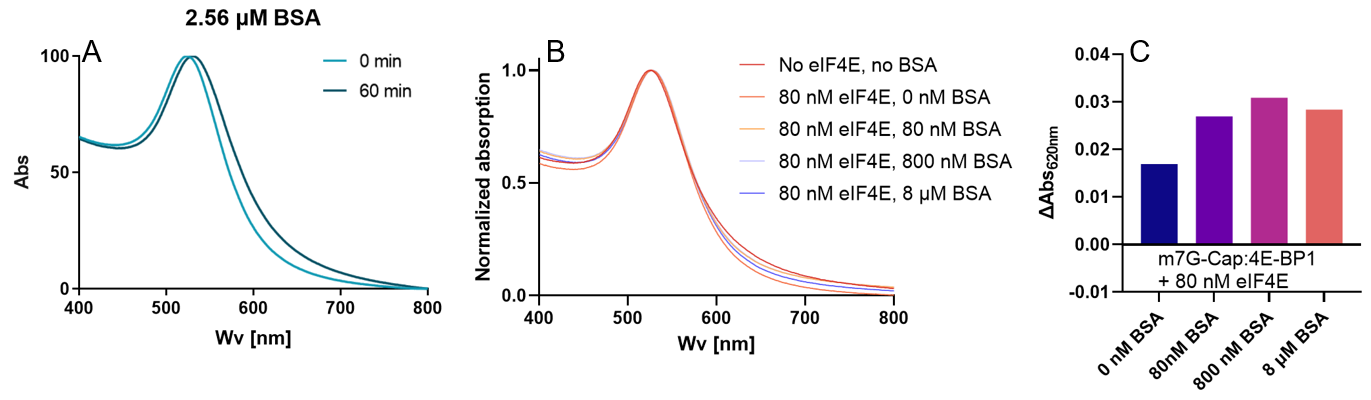


**Figure S13.** Control experiments with BSA protein. A) Absorption changes for 1:1 mixture of m^7^G-cap-TL-AuNPs and 4E-BP1-AuNPS (each at 0.6 nM) after 1 h incubation with BSA protein (2.56 μM). B) UV-Vis spectra of m^7^G-Cap-TL and 4E-BP1 AuNPs conjugate mixture incubated with 80 nM of eIF4E and three different concentrations of BSA for 1h at room temperature. C) Absorption shift upon incubation of AuNPs mixture with eIF4E (80 nM) and three different concentrations of BSA.


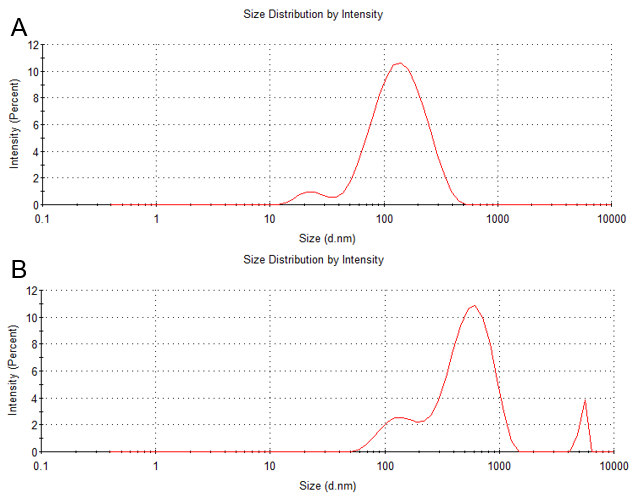


**Figure S14.** DLS-determined size distributions of m^7^G-cap-TL-AuNPs and 4E-BP1-AuNPs 1:1 conjugate mixture. A) Before incubation with eIF4E and B) after 1h incubation with eIF4E (2.56 µM) at room temperature.
